# Case studies on the management of public health events in Africa, Central Asia, South Asia, and the Middle East - the GIBACHT approach

**DOI:** 10.11604/pamj.supp.2021.40.2.34149

**Published:** 2021-12-17

**Authors:** Eva Mertens

**Affiliations:** 1 Bernhard Nocht Institute for Tropical Medicine, Department of Infectious Disease Epidemiology, Bernhard-Nocht-Strasse 74, 20359 Hamburg, Germany,; 2 Global Partnership Initiated Biosecurity Academia for Controlling Health Threats (GIBACHT), Hamburg, Germany

**Keywords:** Case studies, GIBACHT, public health

## Editorial

The Global Partnership Initiated Biosecurity Academia for Controlling Health Threats (GIBACHT) is a multilateral one-year biosafety and biosecurity training programme that has completed seven cohorts since its inception in 2014. GIBACHT is responding to the needs of countries with insufficiently prepared health systems and risk of accidental and deliberate release of infectious agents. The programme is supported by the German Biosecurity Programme, coordinated by the Bernhard Nocht Institute for Tropical Medicine (BNITM) and conducted in partnership with the Robert Koch Institute (RKI), the Swiss Tropical and Public Health Institute (Swiss TPH), and the African Field Epidemiology Network (AFENET). GIBACHT reaches specialists from public health institutions in partner countries in Africa, the Middle East, and South and Central Asia, who play a key role in managing biological incidents. An integral part of the GIBACHT programme is the development and implementation of context-specific case studies covering the topics of biosafety and biosecurity by the GIBACHT fellows. The scenarios of the individual case studies are selected by the fellows based on their experiences and are intended to address the specific needs and requirements of their respective country or professional environment. In line with the “training-of-trainers concept” of the programme, the goal is to implement the case study as a teaching tool at the home institution of the fellow. The development of the case studies is closely monitored and supervised by the GIBACHT consortium and GIBACHT alumni supporting the programme. In the course of the fellowship, the fellows have the opportunity to pilot test their developed case studies with Public Health students invited from the Makerere University of Kampala. This supplement contains five case studies from GIBACHT fellows from Egypt, Ethiopia, Ghana, Iran, Kazakhstan, Kyrgyzstan, Nigeria, Pakistan, Sudan, Tunisia, and Uganda. They consist of the presentation of a biosafety/biosecurity scenario, which the target audience is working through by responding through a set of guided questions in group discussions. The case studies encourage critical thinking and problem-solving, and are versatile tools to be applied in a multitude of teaching situations.

